# Soil Acidobacterial 16S rRNA Gene Sequences Reveal Subgroup Level Differences between Savanna-Like Cerrado and Atlantic Forest Brazilian Biomes

**DOI:** 10.1155/2014/156341

**Published:** 2014-09-15

**Authors:** Elisa C. P. Catão, Fabyano A. C. Lopes, Janaína F. Araújo, Alinne P. de Castro, Cristine C. Barreto, Mercedes M. C. Bustamante, Betania F. Quirino, Ricardo H. Krüger

**Affiliations:** ^1^Cellular Biology Department, Instituto Central de Ciências Sul, Universidade de Brasília (UnB), 700910-900 Brasília, DF, Brazil; ^2^Genomic Sciences and Biotechnology, Universidade Católica de Brasília, 70790-160 Brasília, DF, Brazil; ^3^Ecology Department, Universidade de Brasília (UnB), 700910-900 Brasília, DF, Brazil; ^4^Genetics and Biotechnology Laboratory, Embrapa-Agroenergy, 70770-901 Brasília, DF, Brazil

## Abstract

16S rRNA sequences from the phylum Acidobacteria have been commonly reported from soil microbial communities, including those from the Brazilian Savanna (Cerrado) and the Atlantic Forest biomes, two biomes that present contrasting characteristics of soil and vegetation. Using 16S rRNA sequences, the present work aimed to study acidobacterial diversity and distribution in soils of Cerrado savanna and two Atlantic forest sites. PCA and phylogenetic reconstruction showed that the acidobacterial communities found in “Mata de galeria” forest soil samples from the Cerrado biome have a tendency to separate from the other Cerrado vegetation microbial communities in the direction of those found in the Atlantic Forest, which is correlated with a high abundance of Acidobacteria subgroup 2 (GP2). Environmental conditions seem to promote a negative correlation between GP2 and subgroup 1 (GP1) abundance. Also GP2 is negatively correlated to pH, but positively correlated to high Al^3+^ concentrations. The Cerrado soil showed the lowest Acidobacteria richness and diversity indexes of OTUs at the species and subgroups levels when compared to Atlantic Forest soils. These results suggest specificity of acidobacterial subgroups to soils of different biomes and are a starting point to understand their ecological roles, a topic that needs to be further explored.

## 1. Introduction

Acidobacteria are one of the most abundant phyla in the soil habitats [1]. More than 30% or even 50% of the sequences obtained in 16S rRNA gene clone libraries from soil belong to the phylum Acidobacteria [2] and a great part (4 to 16% transcripts of 16S rRNA from 9 to 31% 16S rRNA genes of Acidobacteria) of these are also physiologically active [3]. Currently, there are 26 acidobacterial subgroups [4], but not all subgroups have cultured representatives. The ubiquitous and abundant presence of Acidobacteria in soils suggests that these microorganisms have an important role in biogeochemical cycles. Acidobacteria may be, for example, essential for maintenance of soil fertility and to enhance vegetation growth, since microorganisms are related to organic matter decomposition and nutrient cycling. However, the answer to these questions still depends on culturing Acidobacteria under laboratory conditions.

Although the number of cultivated Acidobacteria is constantly increasing, only few species have been isolated from soils and marine environments. Currently, there are only 14 described genera, eight belonging to Acidobacteria subgroup 1 (GP1): Acidobacterium [5],* Terriglobus* [6, 7],* Edaphobacter* [8],* Granulicella* [9, 10], “*Acidiphila”* [11],* Telmatobacter* [12],* Acidicapsa* [13], and* Bryocella* [14]. Subgroup 8 is the Class Holophagae and comprises the genera* Holophaga* [15],* Geothrix* [16], and* Acanthopleuribacter* [17]. There is only one genus in culture belonging to subgroup 3 (GP3):* Bryobacter* [18], and recently, two genera from subgroup 4 (GP4) were described:* Blastocatella* [19] and its close relative* Aridibacter* [20]. The genus* Thermotomaculum* is the only cultured representative of subgroup 10 [21]. Although these isolates were obtained from distinct environments ranging from acidic environments [5, 13, 18] to marine hydrothermal vents [21], to the tundra soil [9], they are usually considered to be slow-growing organisms that thrive in oligotrophic habitats.

The analysis of 16S rRNA gene clone libraries and pyrosequencing demonstrated that the abundance of* Acidobacteria* is usually correlated with low values of soil pH [22] and that subgroups GP1, GP4, and GP6 are predominant in soil [1, 23]. These works consider mostly soils from the northern hemisphere and* Acidobacteria* from Brazilian biomes have not been compared yet.

Brazil comprises six biomes: the Atlantic Forest, the Amazon Forest, the Cerrado (the Brazilian Savanna), the Caatinga, the Pampa, and the Pantanal (see Figure S1 in Supplementary Material available online at http://dx.doi.org/10.1155/2014/156341). The Cerrado and the Atlantic Forest are on the list of the world's priorities for conservation efforts because they are biodiversity hotspots [24]. Both of these Brazilian biomes are rapidly being converted to pastures and agricultural fields. The Cerrado is a Savanna-like biome that occurs mostly in the center-west region of country. It is the second largest biome of Brazil, corresponding to 24% of the Brazilian territory (http://www.ibama.gov.br/). In contrast to the inland Cerrado, the Atlantic Forest is a biome that occurs mainly in the Brazilian coast, from the state of Rio Grande do Norte to Rio Grande do Sul.

Aiming to compare the diversity and structure of acidobacterial communities in contrasting Brazilian ecosystems, here we present the analysis of 16S rRNA gene sequences from three works that describe soil acidobacterial communities from the Cerrado (present work, also described in [25]) and the Atlantic Forest [26, 27]. The analysis of soil parameters that may influence the diversity and distribution of Acidobacteria subgroups is also presented. The ecological roles of* Acidobacteria* in soils are not well understood. In this work, we begin to address this topic by correlating specific Acidobacteria subgroups to the soil environment found in the Brazilian biomes Cerrado and Atlantic Forest.

## 2. Material and Methods

### 2.1. Cerrado Soil Sampling and Analysis

Soil samples were collected from the upper 10 cm in areas of Campo sujo (S15° 56′ 54.6′′; WO 47° 52′ 11.7′′), Cerrado denso (S15° 56′ 43.1′′; WO 47° 51′ 26.0′′), Cerrado* sensu stricto* (S15° 57′2.4′′; WO 47° 52′ 32.1′′), and “Mata de galeria” (S15° 57′ 06.0′′; WO 47° 53′ 18.7′′) in October 2006, in the beginning of the rainy season, on the Ecological Reserve of IBGE, DF, Brazil. The physicochemical properties of all soils were analyzed by standard methods (Solo Quimica, Inc., Brazil). Ten sampling points were chosen for each area in which 5 samples were taken. Two composite samples from 5 sampling points were made to represent each vegetation type.

Total DNA of the microbial community present in soil samples was extracted using the kit PowerSoil DNA Isolation Kit (MOBIO Laboratories, Inc.) according to manufacturer's instructions. 16S rDNA PCR using metagenomic DNA from soil as a template was amplified with the bacterial-specific primers 27F (5′ AGAGTTTGA TCMTGGCTCAG 3′) and 1492R (5′ GGYTACCTTGTTACGACTT 3′). The amplified bands were ran in a 1% agarose gel, excised, and cloned into pGEM-T Easy. The clones were stored at −80°C in ELISA plates containing glycerol. The sequencing reactions of the inserts were performed using an automated DNA sequencer ABI PRISM 377 (Applied Biosystems).

### 2.2. Areas Studied

The Cerrado biome is located in Central Brazil and presents a seasonally dry climate with a marked rainfall season (reaching 1,500 mm per year). Its vegetation ranges from grassland to forests [28, 29]. Samples were taken in four different vegetation types. The “Mata de galeria” subarea (MG) diverges from the others for its proximity to water streams.

The Atlantic forest presents ombrophilous vegetation in a gradient that goes from a predominance of shrubs to dense mountain forests that differ according to soil, topography, and local climate [30]. “Serra dos Órgãos” National Park is a mountainous area in the Southeast of Brazil in the state of Rio de Janeiro, that is, part of the “Serra do Mar” mountain range. Both the soil bacterial diversity at “Serra dos Órgãos” National Park (PARNASO) [26] and the soil bacterial community in the “Serra do Mar” near the State of Paraná were described [27]. These areas of coastal Atlantic Forest present the greatest indexes of rainfall precipitation of Brazil that can reach 4,000 mm per year with a humid tropical and subtropical climate without dry season.

The vegetation, the average temperature, and net rainfall are some of the main characteristics that differ between those biomes [30, 31], which may lead to different rates of decomposition of soil organic matter. Soils from both biomes studied are dystrophic, acidic, and weathered. Although the types of soil vary with the vegetation types, Cerrado soil is mostly classified as oxisol, while Atlantic Forest soil is cambisol and litholic neosol (Serra do Mar) or oxisol in lower altitudes (Serra dos Órgãos). Both oxisol and cambisol soils have a high proportion of clay; neosol is a sandy soil.

### 2.3. Construction of Acidobacterial 16S rRNA Gene Database

In order to perform a comparison of acidobacterial communities within different Brazilian biomes, published literature and the GenBank database have been searched for bacterial 16S rRNA gene clone libraries matching the following criteria: (i) communities from Brazilian soil native environments; (ii) clones sequenced by the Sanger method; (iii) universal primers to the same 16S rRNA variable regions. Only two studies met these criteria to compare to our sequences from the Cerrado area (SA): the “Serra dos Órgãos” (SO) National Park study [26] and the “Serra do Mar” (SM) study [27].

SO and SM soils were sampled in different sites of Atlantic Forest vegetation. Six subareas were sampled in SO area and were named S1 to S6 corresponding to the “Bonfim Amarelo,” “Bonfim Vermelho,” “Ajax,” “Torto,” “Campo Úmido,” and “Cavalinho” areas, respectively [26]. Similarly, SM subareas were named from MA01 to MA10, which correspond to areas with decreasing altitude in the “Serra do Mar” mountain range [27]. The Cerrado area is the only one with duplicates for each library as the two works from Atlantic Forests did not have replicates. For this reason, the sequences from replicates samples of Cerrado were merged so that the same standards were used for all areas.

The sequences used in this work were obtained from bacteria present in the soil upper 10 cm [25, 26] or 20 cm [27]. These works used bacteria-specific primers 27F (5′ AGA GTT TGA TCM TGG CTC AG 3′) and 1492R (5′ GGY TAC CTT GTT ACG ACT T 3′) [32] to evaluate diversity.

### 2.4. Comparison of Acidobacterial Communities

To assign 16S rRNA gene sequences to a taxonomic hierarchy, sequences were classified using the 2011 Greengenes' MOTHUR ready-files database of 16S rRNA sequences (http://www.secondgenome.com/go/2011-greengenes-taxonomy/) [33] with the* classify.seqs* function in MOTHUR [34], using 90% of confidence threshold. Sequences assigned by Greengenes [33] as belonging to the phylum Acidobacteria were selected with MOTHUR [34] from the total bacterial sequences databases (HM580079 to HM 581508 and HM563080 to HM563647 from the present study HM063843 to HM063928 from [26]; EF135620 to EF136358 and GU071058 to GU071072 from [27]). Sequence length for the SA and SO database was approximately 450 bp, while for the SM database it was approximately 270 bp. All the libraries from the three areas were normalized to have approximately the same number of sequences. Sequences from the three acidobacterial databases (SO, SM, and SA) were aligned using the program CLUSTALX 2.1 [35]. The alignment was manually proofread and corrected when necessary using Bio Edit (http://www.mbio.ncsu.edu/) [36].

MOTHUR [34] was used to construct the distance matrix clustered at 97% and 85% levels of sequence similarity from the aligned sequences to calculate the nonparametric indexes of richness (i.e., Ace and Chao1) and diversity (i.e., Simpson and Shannon) as well as coverage for each acidobacterial community.

### 2.5. Phylogenetic Analysis

Aligned sequences were clustered into operational taxonomic units (OTUs) using MOTHUR [34]. This program was also used to define shared and unique OTUs between acidobacterial communities of SO, SM, and SA in a Venn diagram for 97% and 85% sequence similarity levels.

To avoid redundancy of sequences, an acidobacterial phylogenetic tree was constructed with only representative sequences from the OTU clusters obtained with MOTHUR using 85% sequence similarity level. All sequences from cultured Acidobacteria were included in the alignment with those representative sequences. The tree was constructed with the software MEGA 5.05 [37], neighbor-joining method, and Jukes-Cantor model of substitution and bootstrap of 1,000.

### 2.6. Statistical Analysis

Principal component analysis (PCA) with a matrix based on variance-covariance and axes representing soil physicochemical characteristics or relative abundance of subgroups of Acidobacteria was performed with Past v.2.16 [38]. Each vector points to the direction in which the respective value increases.

Correlation analysis between physicochemical parameters and relative abundance of each acidobacterial subgroup was performed in the* R* software v2.15.1 [39]. Pearson coefficients were estimated and hypotheses were accepted when *P* values were less than 0.05. All *P* values were adjusted by the BH method [40] and the Bonferroni correction [41]. The BH method is less stringent than the Bonferroni correction.

## 3. Results

Acidobacteria diversity in Brazilian soil samples was analyzed based on 16S rRNA gene clone libraries to assess the shared core of acidobacterial sequences. In addition, differences in subgroups specific to each biome and their characteristics were studied to identify which Acidobacteria groups are selected by each environment.

Selected physicochemical parameters for each area studied have been compiled in Table 1. Considering that all three areas were sampled across different types of vegetation and soils within the same biome, some differences are encountered between libraries of SA area (CS, SS, CD, MG), SO (S1–S6), and SM (MA01–MA10). On average, Atlantic Forest's soils have higher content of Al^3+^, exchangeable cations (Al^3+^ and H^+^), and phosphorus (P) than those found in Cerrado soils (Table 1). Interestingly, “Mata de galeria” (MG) soils in the Cerrado biome have greater values for those parameters than the other soils from the same biome which approximates it to the Atlantic Forest soils' characteristics. All soils described here are acidic; however, in this study soils from the Atlantic Forest have shown pH one order of magnitude lower than the soils from the Cerrado (Table 1).

### 3.1. Acidobacterial Classification

For all soils sampled in the Cerrado and the Atlantic Forest subareas it was found that more than 50% of the sequences were classified as Acidobacteria according to the Greengenes taxonomical classification, except in site 5 (S5) of “Serra dos Órgãos” (SO) bacterial community, as previously described [26]. This phylum was followed by other abundant phyla such as Proteobacteria, Chloroflexi, Verrucomicrobia, Gemmatimonadetes, and Actinobacteria.

From the total bacterial area libraries, 1056, 403, and 449 acidobacterial sequences were obtained for analysis from the Cerrado (SA), the “Serra dos Órgãos” (SO), and the “Serra do Mar” (SM), respectively. To perform all the analyses, we performed a subset of the number of sequences from SA area to normalize to the number of sequences in SO and SM.

**Figure 1:**
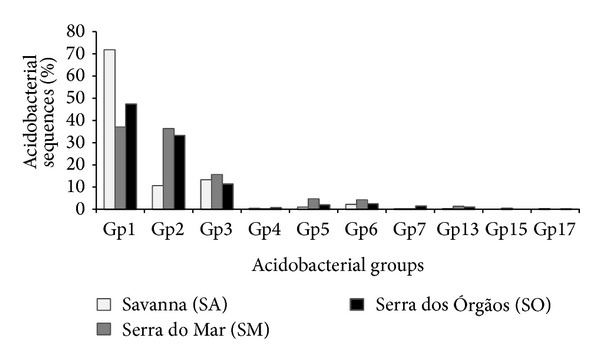
Percentage of acidobacterial groups in relation to the total of acidobacterial sequences in Cerrado (SA, Brazilian Savanna) and Atlantic Forest soils at two sites: “Serra dos Órgãos” (SO) and “Serra do Mar” (SM).

The 16S rRNA gene libraries from the three areas were analyzed according to the number of sequences for each subgroup relative to the total of acidobacterial sequences (Figure 1). GP1 is the predominant acidobacterial group in all bacterial communities studied, being particularly dominant in the Cerrado soil (SA), where it corresponds to 72.4% of the sequences. However, comparatively to the Cerrado (SA) soil, subgroup GP2 is predominant in both Atlantic Forest soils. Specifically for “Serra do Mar,” the proportion of GP1 and GP2 sequences is almost the same, 37.0% and 36.3%, respectively. Finally, GP3 presents a similar percentage of reads for all three soils studied (i.e.; 13% for the Cerrado, 11.4% for the “Serra dos Órgãos,” and 15.6% for the “Serra do Mar”). Sequences from other Acidobacteria groups such as GP4, GP5, GP6, GP7, GP15, and GP17 were also found in some of the soil samples in a lower percentage.

### 3.2. Acidobacterial Structure Communities

**Figure 2:**
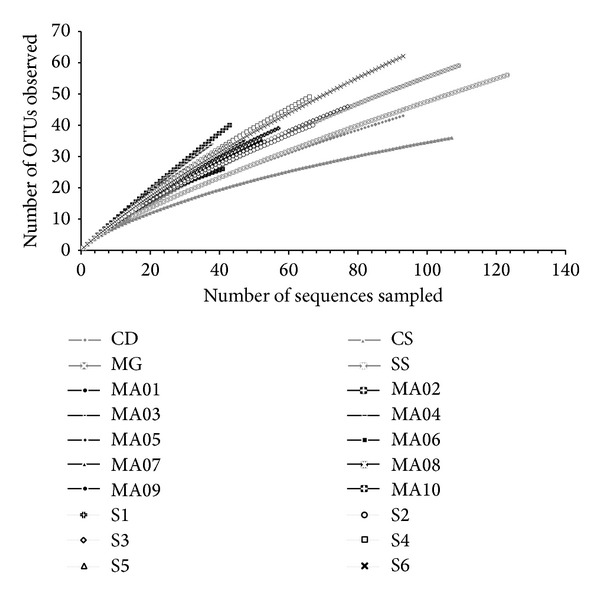
Rarefaction curves for 97% similarity cutoff for acidobacterial 16S rRNA gene library sequences for the following areas and respective subareas. Cerrado (SA): “Mata de galeria” (MG), “campo sujo” (CS), “Cerrado* sensu stricto*” (SS), “Cerrado denso” (CD); “Serra dos Órgãos” (SO): sites 1 to 6 (S1, S2, S3, S4, S5, S6); “Serra do Mar” (SM): MA01, MA02, MA03, MA04, MA05, MA06, MA07, MA08, MA09, MA10.

To compare richness and diversity between the acidobacterial 16S rRNA gene obtained from the three areas studied, MOTHUR was used to calculate Ace and Chao richness indices and Shannon and Simpson diversity indices for all libraries in each one of the three areas (e.g., Cerrado (SA), Atlantic Forest collected at “Serra dos Órgãos” (SO), and Atlantic Forest collected at “Serra do Mar” (SM)). Both the observed and estimated number of OTUs and also the diversity indices to the presumptive level of species (i.e., 97% of similarity) [42] and subgroups (i.e., 85%, considered as the equivalent to the Acidobacteria subgroups, e.g., GP1, GP2, etc.) [43] indicate a greater richness and diversity in the areas of the Atlantic Forest when compared to the Cerrado (Table 2) with great variability in the number of OTUs estimated and diversity indices within libraries from each area (Table S1). Although the difference between “Serra dos Órgãos” and “Serra do Mar” sites is small, the former presents a slightly higher richness and diversity which is indicated by the lower values of coverage. In addition, the rarefaction curve calculated to 97% of similarity indicates that Cerrado (SA), especially the CS subsample from a grassland area, has the lowest richness as it is closer to reaching a plateau (Figure 2). However, the “Mata de galeria” area (MG) of Cerrado has values of observed OTUs similar to the libraries from the Atlantic Forest areas (SO and SM) (Figure 2).

**Figure 3:**
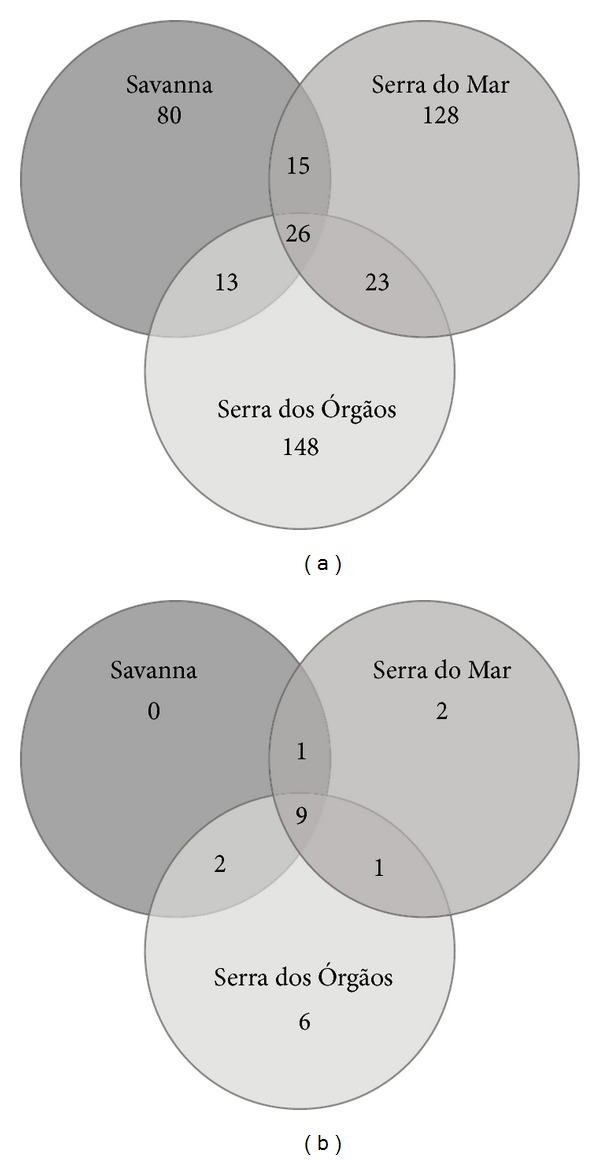
Venn Diagram representing number of OTUs unique to each of the three 16S rRNA gene acidobacterial libraries areas (Cerrado: SA, Atlantic forest “Serra dos Órgãos” site: SO, and Atlantic forest “Serra do Mar” site: SM) and shared between them at 97% (a) and 85% (b) sequence similarity levels.

Venn diagrams using 97% and 85% 16S rRNA gene sequence similarity cutoffs (Figures 3(a) and 3(b)) were constructed in MOTHUR. At both taxonomic levels, OTU richness is lower in the Cerrado, as previously shown in Table 2. The GP1 subgroup is the most abundant in the Cerrado, represented by 29 OTUs at 97% similarity level (98 sequences). This subgroup is represented by 36 OTUs in SM (60 sequences) and 46 OTUs in SO (57 sequences) (Table S2). This is also indicative of the greater acidobacterial richness of the two soils from the Atlantic Forest which also share a greater number of OTUs at 97% when compared to the shared OTUs with the Cerrado soil.

**Figure 4:**
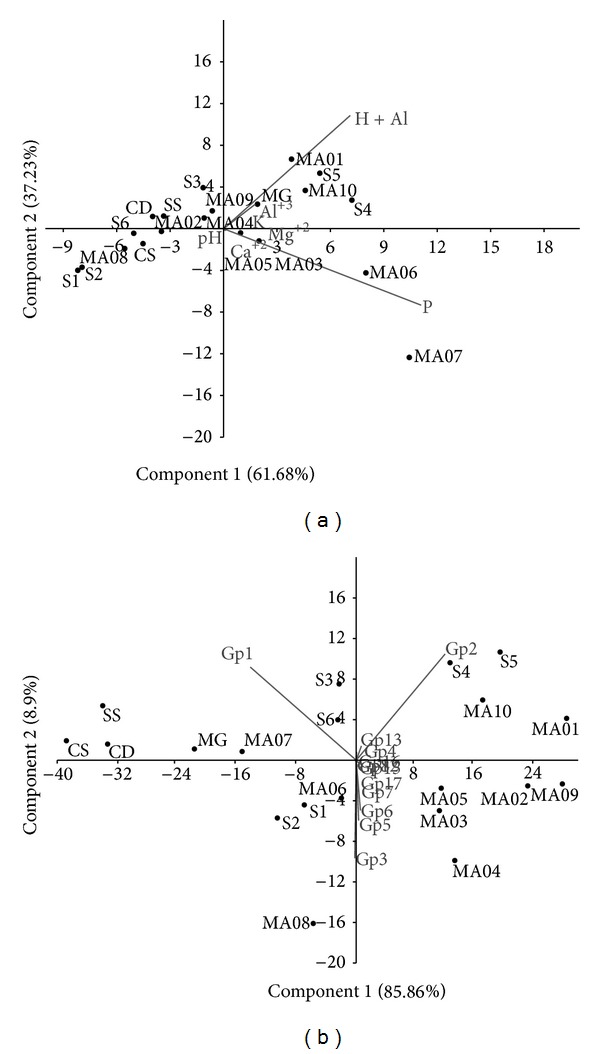
Principal component analysis (PCA) of soil acidobacterial 16S rRNA gene libraries. A matrix of variation-covariation was performed on PAST to analyze acidobacterial 16S rRNA gene sequences from the following areas of Cerrado (SA): “Mata de galeria” (MG), “campo sujo” (CS), “Cerrado* sensu stricto*” (SS), “Cerrado denso” (CD); “Serra dos Órgãos” (SO): sites 1 to 6 (S1, S2, S3, S4, S5, S6); “Serra do Mar” (SM): MA01, MA02, MA03, MA04, MA05, MA06, MA07, MA08, MA09, MA10. (a) Analysis based on the soil physicochemical characteristics for all sites. (b) Analysis based on the relative abundance of subgroups of Acidobacteria for all sites. Each vector points to the direction in which the respective value increases.

Principal component analysis (PCA) when considering physicochemical variables was not able to explain the differences between the acidobacterial libraries from the three areas; we can see that the “Mata de galeria” (MG) vegetation type from Cerrado tends to separate from the other SA libraries and cluster with other SO and SM libraries (Figure 4(a)). GP1 and GP2 abundances were the main explanation vectors to the segregation of areas in the PCA performed with the acidobacterial subgroups abundance (Figure 4(b)). Further, the variance of SA from SO and SM libraries is better explained by both the Al^3+^ and H + Al contents and GP2 abundance (Figures 4(a) and 4(b)).

**Figure 5:**
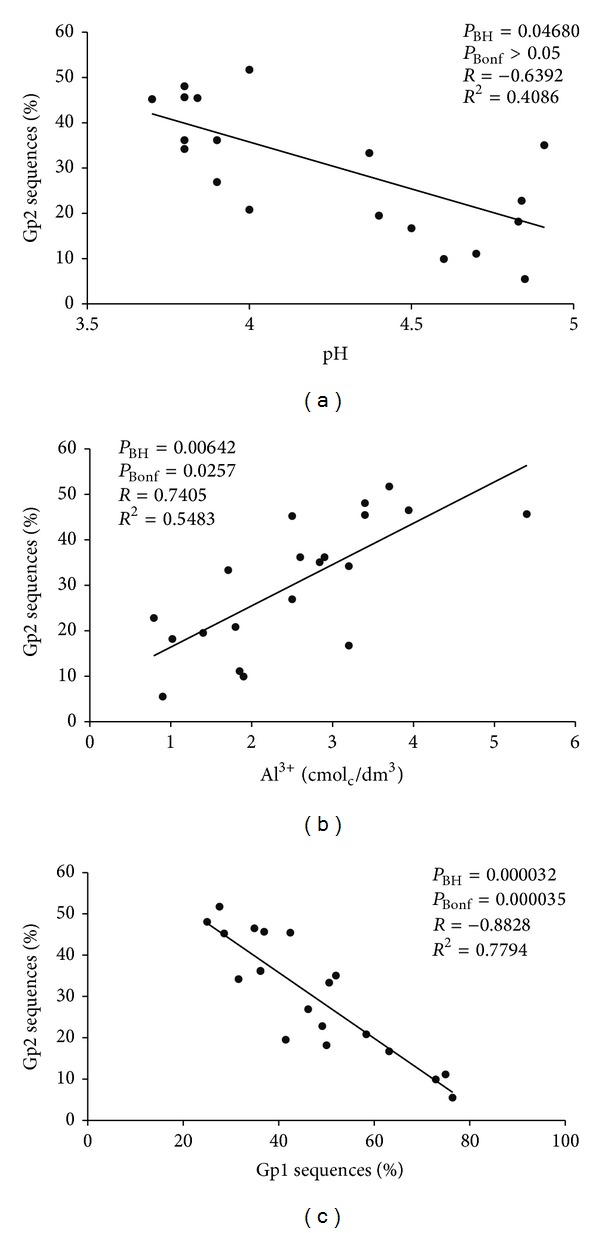
Effect of different factors on GP2 abundance. (a) pH, (b) aluminum, and (c) GP1 abundance on the abundance of GP2 Acidobacteria relative to the total of Acidobacteria. The Pearson coefficient with a corrected *P* value by BH method (*P*_BH_) or Bonferroni correction (*P*_Bonf_) is shown in the upper portion of each panel, as well as the coefficient of correlation (*R*) and determination (*R* ^2^) to show the statistical significance of results.

Pearson coefficient analysis was performed to relate physicochemical parameters and GP2 sequence abundance. Both the BH method and Bonferroni correction were used to correct the *P* value to multiple comparisons. The latter is a conservative method that ensures that error type I (false positive) is not made, although it is more likely to observe the error type II (false negative). In this sense, the BH method was also calculated to be more permissive. The subgroup 2 (GP2) is more abundant in the Atlantic Forest areas and presented a tendency to correlate negatively with pH (Figure 5). It was also found that the amount of GP2 sequences was positively correlated with Al^3+^ content and negatively with that of GP1 sequences (Figure 5).

### 3.3. Acidobacterial Phylogenetic Reconstruction

**Figure 6:**
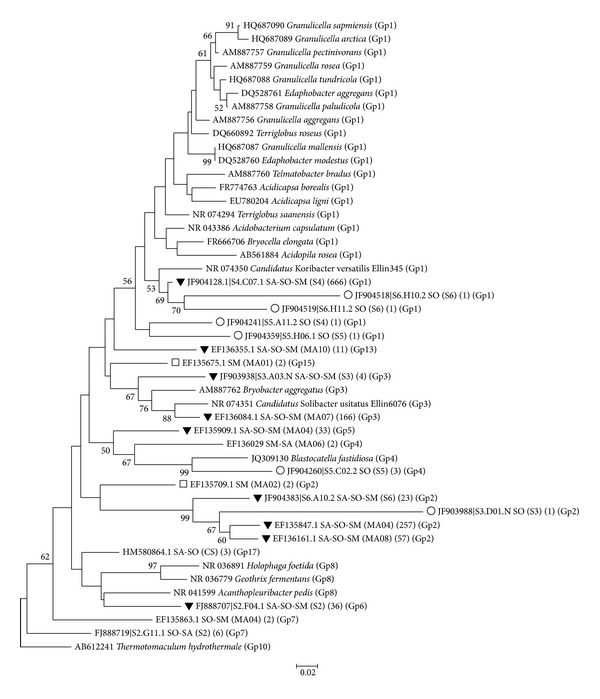
Acidobacterial phylogenetic reconstruction with representative OTUs using 85% similarity cutoff obtained with MEGA 5.05, using the neighbor-joining method, the Jukes-Cantor model of substitution, and a bootstrap of 1,000. Bootstrap values are shown for those branches with >50% support. The scale bar indicates the similarity distance between branches. Blank circles and squares represent exclusive OTUs for “Serra dos Órgãos” and “Serra do Mar,” respectively. Inverted triangles represent OTUs shared by all three areas (Cerrado, “Serra dos Órgãos,” and “Serra do Mar”: SA, SO, SM). Each line in the phylogenetic tree shows first the NCBI accession number for the representative sequence, second the region of the Venn Diagram from which the representative OTU is derived unique from “Serra dos Órgãos” (SO), exclusive from “Serra do Mar” (SM), or shared from two or three areas (i.e. Savanna-Serra dos Órgãos (SA-SO)), third in parenthesis the soil subarea where the representative was sampled (Cerrado (SA): “Mata de galeria” (MG), “campo sujo” (CS), “Cerrado* sensu stricto*” (SS), “Cerrado denso” (CD); “Serra dos Órgãos” (SO): sites 1 to 6 (S1, S2, S3, S4, S5, S6); “Serra do Mar” (SM): MA01, MA02, MA03, MA04, MA05, MA06, MA07, MA08, MA09, MA10), fourth in parenthesis the number of sequences represented by the OTU, and fifth the acidobacterial clusters of subgroups marked according to the name of the group.

A phylogenetic tree was constructed based on representative sequences for each OTU using 85% sequence similarity threshold (i.e., subgroups level) (Figure 6) along with the sequences from all cultivated Acidobacteria.

The phylogenetic reconstruction (Figure 6) was to provide an in-depth analysis of the representative sequences from each region (i.e., shared and exclusive OTUs) of the Venn diagram shown in Figure 3(b). From the 21 OTUs represented in the tree, none were found exclusively in the Cerrado, two OTUs (representing 4 sequences) were exclusive to the Atlantic forest “Serra do Mar,” and six OTUs were exclusive to the Atlantic forest “Serra dos Órgãos” (Figure 6, blank squares and circles, resp.). There is only one GP15 OTU representative from “Serra do Mar” that is the only GP15 sequence described in this work, and the other 2 sequences are distributed in the GP2 group. “Serra dos Órgãos” presented the greatest diversity as there is one OTU represented in the GP2 clade, one in GP4, and the other four represented in GP1.

## 4. Discussion

Comparative analysis of the 16S rRNA gene sequences present in the Cerrado and the Atlantic Forest has shown that nine phyla are the most abundant in almost all soil types. Specifically, these commonly abundant phyla are Proteobacteria, Acidobacteria, Actinobacteria, Verrucomicrobia, Bacteroidetes, Chloroflexi, Planctomycetes, Gemmatimonadetes, and Firmicutes [1]. While in Cerrado soils Acidobacteria corresponded to more than 50% of the sequences, in the Atlantic forest at “Serra dos Orgãos” it accounted for between 29% and 54% of sequences depending on the specific site [26] and 63% of sequences at “Serra do Mar” [27]. The abundance of acidobacterial sequences present in the 16S rRNA library was similar to that previously reported using pyrosequencing technique also by our group [44].

Even though Acidobacteria are commonly described in microbial soil diversity studies, their role in biogeochemical cycling and their influence on the microbial community structure are mostly unknown. The present work presented the acidobacterial community for three areas from two Brazilian biomes. Here a greater abundance of GP2 in soils from Atlantic Forests and “Mata de galeria” areas from the Cerrado was demonstrated, suggesting an influence of the environment on the abundance of acidobacterial subgroups. GP2 is not commonly described in the literature [1, 22, 23, 45] and in Brazilian soils it has only been described in a study of metagenomic DNA libraries for Cerrado soil [46]. This might be valuable information for future research that aims to isolate organisms from the GP2 subgroup.

The numerical abundance of Acidobacteria in soil may reflect this phylum's contribution to decomposition, an important ecological function suggested for Acidobacteria. This role has been suggested due to the presence of several genes that may be part of carbohydrate polymer-degrading pathways [47–49] described in most of Acidobacteria isolates, despite the fact that they are oligotrophic organisms negatively correlated with organic matter in soil [50].

Alignment of all the acidobacterial sequences from Cerrado (present work), Atlantic forest at ‘‘Serra dos Órgãos (SO)” [26], and ‘‘Serra do Mar (SM)” [27] and taxonomical analysis showed the predominance of GP1 in all soils, followed by GP2 and GP3. The prevalence of GP1 and GP2 was previously reported by Bruce et al. [26] in SO. GP2 is more abundant in Atlantic Forest soils in comparison to Cerrado soil and these sequences promoted the difference between the Cerrado and Atlantic forest as shown in the PCA (Figure 4(b)).

GP1 of Acidobacteria is the most mentioned subgroup in the literature as its members are the most readily culturable [1]. Acidobacteria subgroups GP1, GP2, and GP3 were relatively more abundant in all soils studied. It must be noted that the high abundance of GP1, GP2, and GP3 in Atlantic forest and Cerrado soil samples (i.e., phylogenetic redundancy within each group) does not necessarily imply functional redundancy [51]. On the other hand, if Acidobacteria within a subgroup have similar ecological roles, functional redundancy could be important as a buffer to environmental disturbances (i.e., “insurance hypothesis” [52]).

The results obtained by PCA (Figure 4) showed that “Cerrado* sensu stricto*,” “Cerrado denso,” and “Campo sujo” have similar acidobacterial communities. In contrast, “Mata de galeria” was more distant from the other areas in the PCA analysis. These results may be related to the physicochemical properties of soil, as the “Mata de galeria” soil is the most different from the Cerrado soils studied, presenting higher levels of organic carbon content, available phosphorus, calcium, and lower clay content [44]. Further, the acidobacterial community profile may be influenced not only by the soil characteristics but also by vegetation cover. The acidobacterial community profile and soil characteristics are intertwined factors that influence each other. Sequences from Cerrado (SA) and “Serra do Mar” (SM) are grouped separately. Only acidobacterial sequences from the areas sampled in “Serra dos Órgãos” did not cluster with each other (Figure 4(b)).

Both areas of the Atlantic Forest showed greater acidobacterial richness and diversity when compared to the Cerrado soil either to the presumptive level of species (97% sequence similarity) or to the subgroups level (85% sequence similarity). “Serra dos Órgãos” presents a slightly higher richness and diversity (Table 2) than “Serra do Mar” which is consistent with the greater number of exclusive OTUs showed in the Venn diagrams (Figures 3(a) and 3(b)).

In the phylogenetic reconstruction (Figure 6), the most abundant OTU, composed by 666 sequences, that was found in the acidobacterial libraries from the three areas (SA-SO-SM), clustered together with “*Candidatus* Koribacter versatilis” (also known as Ellin345) [47]. This OTU representative sequence showed 93% similarity with the “*Candidatus* Koribacter versatilis” 16S rRNA gene sequence which is an aerobic and heterotrophic bacterium that uses carbon sources with different levels of complexity. These are common characteristics to other genera of subgroup 1 (GP1) such as Acidobacterium [5],* Terriglobus*,* Acidicapsa*,* Acidiphila*,* Telmatobacter*,* Bryocella, Granulicella*, and* Edaphobacter*.

The other two comprehensive clades observed in the phylogenetic tree (Figure 6) are those of GP2 and GP3. However, there is no cultured representative of GP2. One OTU representative of GP3 showed 93% similarity with the sequence of* Candidatus Solibacter usitatus* also known as Ellin6076 [47], which is also an heterotrophic organism, with a genome that suggests ability to degrade different types of carbohydrate substrates and that also appears to be capable of nitrate and nitrite reduction.* Acidobacterium capsulatum*, Ellin345, and Ellin6076, in GP1, appear to be resistant to water stress [47].

Recently,* Blastocatella fastidiosa*, an heterotrophic and aerobic Acidobacteria from GP4, has been isolated [19] and clustered with other OTUs from the present study, showing 90% similarity with one OTU exclusive from the libraries of SO area. In contrast to the prevalence of GP1, GP4, and GP6 Acidobacteria from temperate forests of the northern hemisphere [1, 53], in the Brazilian soils here studied, there was only a small percentage of GP4 sequences (Figure 1). This may be due to the low pH of these soils, as GP4 abundance is positively correlated to pH [22], even though the only known isolate from GP4,* Blastocatella fastidiosa*, was isolated from a Savanna soil with a moderate acidic pH (i.e., close to 6.0) in Namibia.

Environmental characteristics are better predictors of differences between communities than geographic distance [45]. In this study, soil physicochemical parameters derived from the vegetation may be one of the factors affecting acidobacterial diversity, as in both the PCA and Venn diagrams, acidobacterial sequences from the two Atlantic forest soils clustered close to each other and to sequences from the Savanna “Mata de galeria.” In the principal component analysis (Figure 4(a)) the* x*-axis explained most of the differences of those soils grouping many of the Atlantic Forest subareas and the “Mata de galeria” Cerrado subarea (MG) by presenting higher contents of aluminum, exchangeable cations (Al^3+^ and H^+^), and phosphorus. On the other hand, pH explained the clustering of all other subareas of Cerrado with some Atlantic Forest sites.

Principal component analysis using the abundance of subgroups as factors shows a similar grouping of subareas explained mostly by the abundance of GP1 and GP2 (Figure 4(b)). According to the Pearson coefficient (Figure 5), the abundance of GP2 presents a significant (*R* = 0.7405) positive correlation with high soil aluminum content. A similar correlation between high soil aluminum content and total acidobacterial abundance has been reported for the Amazon Forest [54]. This is an important result as 50% of the world's potentially arable soils are acidic (especially in the tropics and subtropics) which leads to aluminum solubility increasing Al^3+^ levels, a condition that is toxic to crops [55]. The data presented here suggest that GP2 Acidobacteria may have some metabolic tolerance to aluminum in soil.

Some soil parameters may not affect the role of the acidobacterial community but instead influence the structure of specific subgroups [23]. This may be the reason for the observed negative correlation found for the abundance of GP1 and GP2 (Figure 5). One other study had already presented an opposite trend of abundance between GP1 and GP4. GP4 abundance tends to increase with increasing soil pH, suggesting different physiologies for members of subgroups GP1 and GP4 [19], which may also be the case for GP1 and GP2 in the present study.

In this work we performed the characterization of the acidobacterial communities in three different sites of two Brazilian biomes. The ubiquitous distribution of Acidobacteria hides other layers of complexity as different phylotypes may be exclusive to a particular area. The diversity previously observed when whole bacterial communities were studied was here observed within a single phylum. It is interesting to point out that the abundance and diversity of sequences within Acidobacteria was enough to separate areas and subareas from Atlantic forest and Cerrado. Further, careful analysis was able to reveal similarities between Cerrado “Mata de galeria” and Atlantic forest, which was also associated with high abundance of GP2. These findings are only the beginning for understanding the ecological role of Acidobacteria in the Atlantic forest and Cerrado biomes. As a final note, it should be mentioned that given the high abundance of Acidobacteria in soils in general, this group will likely be a source of enzymes and other products of biotechnological interest when soil metagenomic libraries are screened. An example of this potential is the recent finding of a new lipase from an Acidobacteria [56] derived from an Atlantic forest soil metagenomic library [27].

As no cultured representatives have been described yet for GP2, effort should be made to isolate organisms from this acidobacterial subgroup possibly with methods as the one described for the Cerrado [57]. This would allow genomic surveys and insights into metabolic functions or testing hypotheses of resistance to aluminum and growth inhibition between GP1 and GP2 Acidobacteria.

## Supplementary Material

Brazilian map depicting all six biomes in the South America map are represented in Figure S1. Blank triangle, circle and square symbols represent areas sampled for “Serra dos órgãos”(SO), “Serra do Mar” (SM) and “Cerrado” (SA), respectively.
We would like to add the legends to the tables in supplementary material:
Table S1. Richness and diversity indexes of acidobacterial databases for all libraries separately (97% and 85% 16S rRNA gene sequence similarity cutoffs). Table S2. Unique and shared OTUs and sequences according to acidobacterial subgrouping, using 97% as a 16S rRNA gene sequence similarity cutoff.

## Figures and Tables

**Table 1:** Comparison of physicochemical properties of selected soils from all libraries of each acidobacterial area: Cerrado (SA): “Mata de galeria” (MG), “campo sujo” (CS), “Cerrado *sensu stricto*” (SS), “Cerrado denso” (CD); “Serra dos Órgãos” (SO): sites 1 to 6 (S1, S2, S3, S4, S5, and S6); “Serra do Mar” (SM): MA01, MA02, MA03, MA04, MA05, MA06, MA07, MA08, MA09, and MA10.

Areas	Subareas	pH	Al^+3^ (cmol_c_/dm^3^)	H + Al (cmol_c_/dm^3^)	Ca^+2^ (cmol_c_/dm^3^)	Mg^+2^ (cmol_c_/dm^3^)	P (mg/dm^3^)	K (cmol_c_/dm^3^)
SA (Savanna)	CD	4.60	1.90	10.10	0.20	0.10	0.75	0.09
	CS	4.85	0.90	7.80	0.20	0.10	1.75	0.08
	MG	4.50	3.20	14.15	0.50	0.10	5.00	0.14
	SS	4.70	1.85	10.50	0.20	0.10	1.25	0.10

SM∗ (Atlantic Forest)	MA01	4.00	3.70	18.80	0.60	0.30	4.20	0.20
	MA02	3.70	2.50	9.00	0.30	0.20	1.90	0.09
	MA03	3.90	2.60	11.30	0.30	0.20	7.00	0.11
	MA04	3.80	3.20	11.30	0.30	0.20	3.20	0.11
	MA05	3.80	2.90	11.30	0.30	0.20	5.70	0.12
	MA06	3.90	2.50	12.10	0.50	0.30	13.70	0.24
	MA07	4.00	1.80	6.70	0.60	0.50	20.20	0.08
	MA08	4.40	1.40	6.70	0.90	0.80	1.10	0.12
	MA09	3.80	3.40	12.10	0.30	0.20	3.20	0.13
	MA10	3.80	5.40	16.30	0.30	0.20	6.50	0.14

SO∗∗ (Atlantic Forest)	S1	4.84	0.79	3.56	0.08	0.06	0.09	0.03
	S2	4.83	1.02	3.90	0.33	0.03	0.12	0.11
	S3	4.91	2.84	14.22	0.09	0.07	2.11	0.15
	S4	3.84	3.40	17.50	0.22	0.11	9.15	0.12
	S5	4.58	3.94	18.50	0.17	0.14	6.30	0.23
	S6	4.37	1.71	8.16	0.05	0.04	0.74	0.04

Savanna (SA): “Cerrado denso” (CD), “campo sujo” (CS), “Mata de galeria” (MG), “Cerrado *sensu stricto*” (SS).

∗Serra do Mar (SM): MA01, MA02, MA03, MA04, MA05, MA06, MA07, MA08, MA09, and MA10 [27].

∗∗Serra dos Órgãos (SO): sites 1 to 6 (S1, S2, S3, S4, S5, and S6) [26].

**Table 2:** Number of sequences observed and estimated richness (Ace and Chao1), diversity (Simpson and Shannon), and Good's Coverage indexes for the tree acidobacterial areas with libraries combined, using 97% and 85% 16S RNA gene sequence similarity cutoffs.

Area sampled	Similarity cutoff	Nseqs^1^	Sobs^2^	Chao	Ace	Shannon	Simpson	% coverage
Savanna (SA)	97%	432	134	321.05	314.59	3.78	0.063	79.86
Serra do Mar (SM)	97%	443	192	354.03	483.82	4.81	0.011	75.17
Serra dos Orgãos (SO)	97%	403	210	435.07	667.94	4.95	0.010	65.76
Savanna (SA)	85%	432	12	17	25.91	0.99	0.549	98.84
Serra do Mar (SM)	85%	443	13	14	18.13	1.69	0.243	99.32
Serra dos Orgãos (SO)	85%	403	18	28.5	38.31	1.64	0.292	98.26

^1^Number of sequences; ^2^sequences observed.
