# Supplementary material for: Soil Acidobacterial 16S rRNA Gene Sequences Reveal Subgroup Level Differences between Savanna-Like Cerrado and Atlantic Forest Brazilian Biomes
Source: Int J Microbiol. 2014 Sep 15;2014:156341. doi: 10.1155/2014/156341 (PMC4181792; doi:10.1155/2014/156341)
Supplement: Supplementary file 1 — Brazilian map depicting all six biomes in the South America map are represented in Figure S1. Blank triangle, circle and square symbols represent areas sampled for “Serra dos órgãos”(SO), “Serra do Mar” (SM) and “Cerrado” (SA), respectively. We would like to add the legends to the tables in supplementary material: Table S1. Richness and diversity indexes of acidobacterial databases for all libraries separately (97% and 85% 16S rRNA gene sequence similarity cutoffs). Table S2. Unique and shared OTUs and sequences according to acidobacterial subgrouping, using 97% as a 16S rRNA gene sequence similarity cutoff. [file 156341.f1.pdf]

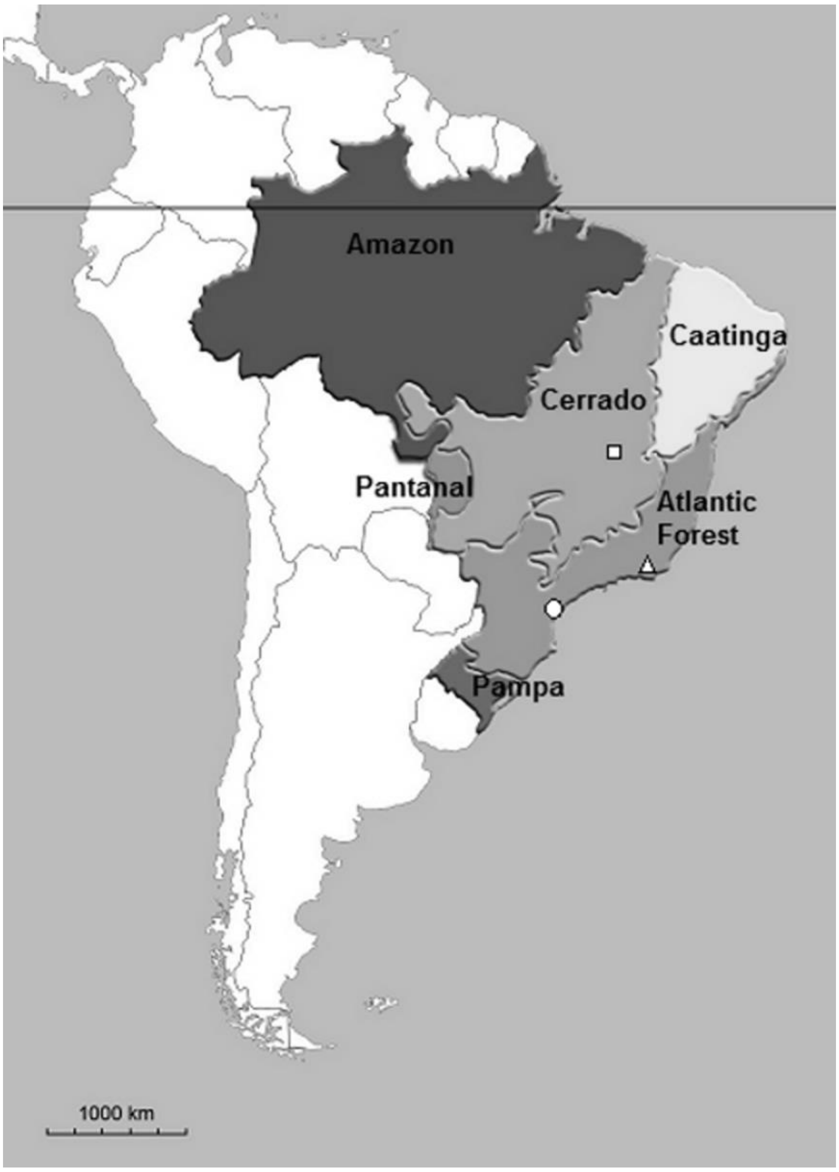

Amazon
Caatinga
Cerrado
Pantanal
Atlantic
Forest
Pampa
1000 km

Area sampled	Subarea	Similarity cutoff	Nseqs[1]	Sobs[2]	Chao	Ace	Shannon	Simpson
SA (Savanna)	CD	97%	93	43	142.2	283.9	3.235	0.055
	CS	97%	107	36	71.0	96.0	2.900	0.093
	MG	97%	109	59	149.3	386.3	3.570	0.051
	SS	97%	123	56	213.7	371.9	3.318	0.068
SM (Serra do Mar)	MA01	97%	29	24	52.5	69.6	3.128	0.012
	MA02	97%	43	41	288.0	440.8	3.697	0.002
	MA03	97%	45	32	60.9	63.1	3.371	0.016
	MA04	97%	38	35	159.0	221.7	3.528	0.004
	MA05	97%	47	35	143.8	490.1	3.362	0.026
	MA06	97%	52	35	60.3	70.3	3.430	0.019
	MA07	97%	48	34	99.0	103.3	3.382	0.020
	MA08	97%	41	27	44.0	48.1	3.181	0.023
	MA09	97%	52	35	93.5	276.6	3.328	0.032
	MA10	97%	48	33	52.1	58.7	3.398	0.017
SO (Serra dos Orgãos)	S1	97%	57	39	84.1	295.7	3.444	0.029
	S2	97%	67	41	118.5	299.5	3.434	0.031
	S3	97%	77	46	112.0	110.0	3.495	0.040
	S4	97%	66	48	165.2	165.3	3.729	0.014
	S5	97%	43	33	63.7	74.7	3.427	0.012
	S6	97%	93	64	170.9	179.3	3.934	0.019

[1] Number of sequences; [2] Sequences observed

Savanna (SA) - 'Cerrado denso' (CD), 'campo sujo' (CS), 'mata de galeria'(MG), 'Cerrado *sensu stricto* ' (SS)

'Serra dos Órgãos' (SO) - sites 1 to 6 (S1, S2, S3, S4, S5, S6)

'Serra do Mar' (SM) - MA01, MA02, MA03, MA04, MA05, MA06, MA07, MA08, MA09, MA10

Subgroups	SAV	SM	SO	SAV-SO	SAV-SM	SM-SO	SAV-SM-SO
Gp1	29	36	46	4	6	6	20
Gp2	18	46	57	5	5	9	3
Gp3	24	19	28	2	4	4	2
Gp4	1	1	2				
Gp5	1	5	2			2	1
Gp6	5	13	4			2	
Gp7		1	5	1			
Gp13	1	5	4				
Gp15		2					
Gp17	1			1			
OTUs Total*	80	128	148	13	15	23	26

* 97% Similarity cutoff

Savanna (SA); 'Serra do Mar' (SM); 'Serra dos Órgãos' (SO)

Subgroups	SAV	SM	SO	SAV-SO	SAV-SM	SM-SO	SAV-SM-SO
Gp1	98	60	57	19	20	20	397
Gp2	24	86	85	17	17	52	58
Gp3	35	30	32	10	30	22	11
Gp4	1	1	3				
Gp5	1	7	2			16	7
Gp6	7	16	4			9	
Gp7		1	5	2			
Gp13	1	6	4				
Gp15		2					
Gp17	1			2			
Number of reads	168	209	192	50	67	119	473

Savanna (SA); 'Serra do Mar' (SM); 'Serra dos Órgãos' (SO)
